# Intracellular Calcium Dysregulation by the Alzheimer’s Disease-Linked Protein Presenilin 2

**DOI:** 10.3390/ijms21030770

**Published:** 2020-01-24

**Authors:** Luisa Galla, Nelly Redolfi, Tullio Pozzan, Paola Pizzo, Elisa Greotti

**Affiliations:** 1 Department of Biomedical Sciences, University of Padua, 35131 Padua, Italy; luisa.galla84@gmail.com (L.G.); nelly.redolfi@unipd.it (N.R.); tullio.pozzan@unipd.it (T.P.); elisa.greotti@cnr.it (E.G.); 2 Neuroscience Institute, National Research Council (CNR), 35131 Padua, Italy; 3 Venetian Institute of Molecular Medicine (VIMM), 35131 Padua, Italy

**Keywords:** presenilins, calcium dysregulation, Alzheimer’s disease, SOCE, genetically encoded calcium indicators

## Abstract

Alzheimer’s disease (AD) is the most common form of dementia. Even though most AD cases are sporadic, a small percentage is familial due to autosomal dominant mutations in amyloid precursor protein (*APP*), presenilin-1 (*PSEN1*), and presenilin-2 (*PSEN2*) genes. AD mutations contribute to the generation of toxic amyloid β (Aβ) peptides and the formation of cerebral plaques, leading to the formulation of the amyloid cascade hypothesis for AD pathogenesis. Many drugs have been developed to inhibit this pathway but all these approaches currently failed, raising the need to find additional pathogenic mechanisms. Alterations in cellular calcium (Ca^2+^) signaling have also been reported as causative of neurodegeneration. Interestingly, Aβ peptides, mutated presenilin-1 (PS1), and presenilin-2 (PS2) variously lead to modifications in Ca^2+^ homeostasis. In this contribution, we focus on PS2, summarizing how AD-linked PS2 mutants alter multiple Ca^2+^ pathways and the functional consequences of this Ca^2+^ dysregulation in AD pathogenesis.

## 1. APP, PS1, and PS2 Physiopathology: Focus on Alzheimer’s Disease

Alzheimer’s disease (AD) is an age-related neurodegenerative disorder, considered the most common cause of dementia. According to the original 1984 diagnostic criteria, and the revised clinical criteria evaluated by the National Institute on Aging-Alzheimer’s Association (NIA-AA) in 2011 [1], AD is characterized by a cognitive decline (i.e., memory impairment, language, and visuospatial function deterioration) that culminates in the loss of abilities necessary to perform basic daily life activities [2,3,4]. Even if new clinical criteria can help to perform AD diagnosis in living patients [5,6], most commonly, this is performed on post-mortem brain tissues, by evaluating the presence of senile plaques, formed by extracellular deposits of amyloid β (Aβ) peptides, and intracellular neurofibrillary tangles (NFT) of the microtubule-binding protein tau [7]. Based on the time of onset, AD is sub-classified in Early-Onset AD (EOAD), indicating patients with an onset before 65 years of age, or Late-Onset AD (LOAD), when the disease begins after 65 years of age [8]. From a genetic point of view, AD can be divided in Sporadic AD (SAD) and Familial AD (FAD) [8,9,10,11], depending on the absence or presence of specific genetic lesions, respectively. 

## 2. Sporadic Alzheimer’s Disease (SAD)

The most common form of AD (with more than 90% of cases) is sporadic, without any specific familial link. Genetic factors, as well as environment and lifestyle, may contribute to its onset. It is usually related to aging as it begins after the age of 60–65 and its frequency increases with age. Among the risk factors, *APOE* gene polymorphisms are the best characterized. *APOE* encodes an apolipoprotein that has a prominent role in cholesterol homeostasis. It exists in different polymorphic forms, and the Epsilon 4 (ε4) form correlates with a major risk of AD, both in homozygosis and heterozygosis [11]. Moreover, *APOE* ε4 increases AD risk in combination with type 2 diabetes [12]. Recently, the P86L polymorphism in *CALHM1* (codifying for a newly discovered Ca^2+^ channel [13]) has been included as a risk factor for SAD [13]. Moreover, it has been suggested that the *CALHM1* P86L polymorphism may modulate AD onset in conjunction with that of *APOE* [14]. Importantly, *CALMH1* polymorphism is reported to impact on the Ca^2+^ permeability of CALHM1 channels [13], suggesting a key role for Ca^2+^ signaling in AD pathogenesis. However, different studies have also argued against this proposal [15,16].

Vascular diseases, traumatic brain injuries, epilepsy, depression, hyperlipidemia have also been considered comorbidity factors for AD. Among lifestyle factors, physical activity, sleep disturbance, and diet could modify AD development and progression [17]. In this context, the Latent Early-life Associated Regulation (LEARn) model hypothesized that exposure to stressors, such as heavy metals, early in life perturbs gene regulation in a long-term fashion, leading to the pathology later in life [18,19,20,21]. Other studies proposed the “mitochondrial cascade hypothesis” [22,23] that correlates age-associated mitochondrial defects with AD histological changes (i.e., Aβ production), placing mitochondrial dysfunctions, in particular, an increased mitochondrial ROS production, at the basis of SAD onset [24].

## 3. Familial Alzheimer’s Disease (FAD)

FAD contributes to 2–3% of all AD cases and is characterized by the early onset of symptoms (age < 65 years) and a positive familial history of dementia in the last three generations, with an autosomal dominant transmission [25]. The genetic factors involved include mutations in amyloid precursor protein (*APP*), presenilin-1 (*PSEN1*), and presenilin-2 (*PSEN2*) genes [10,26]. Even if FAD has some unique characteristics with respect to SAD (i.e., earlier age of onset of symptoms and a positive familial story for dementia), they present common clinical features, including neuronal loss and brain Aβ deposition [26]. The overlapping clinical phenotype of SAD and FAD, and the lack of reliable animal models of SAD, justifies the use of FAD-based mouse models as experimental tools to investigate AD pathogenesis.

### 3.1. Amyloid Precursor Protein

As its discovery in 1987, the functions of APP and its cleavage products have been subjected to intense investigations stimulated by the seminal finding that 40/42 amino acid fragments of APP, called Aβ peptides, are abundant in brain amyloid plaques of AD patients. Human APP is a member of the APP family of conserved type I membrane proteins, located at the plasma membrane (PM), which also includes APP-like proteins 1 and 2 (APLP1 and APLP2). The human *APP* gene is located on chromosome 21 (21q21.3), contains at least 18 exons [27,28], and undergoes tissue-specific splicing, generating different isoforms that encode proteins of different length, ranging from 365 to 770 amino acids [29]. The functional significance of this apparent tissue-specific alternative splicing is still poorly understood [30]. 

In mammals, important insights into APP functions have come from genetic analyses. *APP* knockout (KO) mice are viable, but they have reduced body weight and locomotor activity, disturbed forelimb strength and gliosis, reduced spine numbers, brain mass, and altered long-term potentiation (LTP) response [31]. Instead, APP overexpression in transgenic (tg) mice increases spine density, alters LTP responses and performance in the Morris water maze, reduces brain weight, induces defects in both axonal growth/white matter and axonal transport [32]. Taken together, these data support a role for APP in cellular growth and apoptosis, synapse formation, and maintenance and in neuronal migration in early embryogenesis, by itself or in association with other intracellular proteins (reviewed in [33,34]).

The coverage of such a wide range of functions is partially guaranteed by both post-translational modifications and intracellular proteolytic cleavages that APP undergoes in the constitutive secretory pathway [35,36]. Of note for AD pathogenesis, APP is proteolytically cleaved by α-, β-, and γ-secretases, a class of membrane-bound aspartyl proteinases. APP processing can follow two different pathways: the non-amyloidogenic and the amyloidogenic ones (for reviews, see [37,38]). In the non-amyloidogenic pathway, APP is first cleaved by α-secretase, which yields the soluble sAPPα and leaves the C-terminal fragment (CTFα) in the PM. CTFα is cleaved by γ-secretase producing a soluble extracellular peptide (p3) and the APP intracellular domain (AICD). In the amyloidogenic pathway, BACE1 (β-secretase) cleaves APP to release a soluble fragment (sAPPβ) and generates a membrane-inserted C-terminal peptide, called CTFβ. CTFβ is further cleaved by γ-secretase, resulting in the production of AICD and, extracellularly, Aβ peptides [39]. The γ-secretase cleavage activity is not precise and can produce Aβ peptides of different lengths, ranging from 49 to 38 amino acids. Aβ40 is the most abundant product, while Aβ42, more hydrophobic and more prone to oligomerization, represents only about 10% of total products. 

It is worth mentioning that FAD-APP mutations are mainly localized near the sites of secretase cleavage, promoting the amyloidogenic pathway. These considerations, together with the fact that mutations inside the Aβ peptide sequence favor its aggregation, and that APP overexpression (e.g., in Down Syndrome, due to trisomy 21) is associated with EOAD symptoms, give strong genetic support to the amyloid cascade hypothesis for AD pathogenesis. This proposes that AD-related neurodegeneration is due to a sequence of events generated by the altered processing of APP and subsequent production of Aβ42 peptides that, oligomerizing, form the initial nucleus for subsequent Aβ40 deposition, leading to the formation of fibrils found in brain amyloid plaques [40,41]. A crucial player in this pathway is γ-secretase that releases Aβ peptides from the membrane. Different mechanisms have been proposed to explain the pathogenic activity of Aβ, such as alterations in Ca^2+^ homeostasis, mitochondrial, and energy metabolism dysfunctions, formation of cationic channels in the PM, oxidative stress due to ROS overproduction, cytoskeleton, and axonal transport alterations, inflammatory processes, increased susceptibility to pro-apoptotic and pro-necrotic stimuli [42,43,44,45,46]. Of note, it has been proposed that Aβ soluble oligomers, and not the insoluble fibrils, are the toxic forms causing synaptic dysfunctions [47].

### 3.2. Presenilins

*PSENs* were first identified in 1995 looking for genes responsible for early-onset, autosomal dominant forms of FAD [48,49]. A few years after their discovery, it was shown that *PSEN*s encode proteins that, acting as aspartyl proteases, sustain γ-secretase cleavage of APP to produce Aβ peptides [50,51]. *PSEN* genes are highly conserved during evolution having homologs in organisms as distant as *Caenorhabditis elegans* [52], *Drosophila melanogaster* [53], and lower chordates [54]. 

*PSEN1* and *PSEN2* mRNA are ubiquitously and equally expressed in different human and mouse tissues, including brain regions [55], with the highest expression levels in the hippocampus and cerebellum. They are mainly expressed in neurons, but detectable also in glial cells [56,57]. *PSEN1*, on chromosome 14 (14q24.3) in humans, encodes presenilin-1 (PS1) while *PSEN2*, on chromosome 1 (1q42.2), encodes presenilin-2 (PS2). Both PS1 and PS2 are 50-kDa polytopic transmembrane (TM) proteins that share an identity of about 65% and are mainly localized at the endoplasmic reticulum (ER) and Golgi Apparatus (GA) membranes but also, although less abundantly, at PM and endosomes [58]. More recently, PSs have been reported to be enriched at mitochondria-associated membranes (MAM; see below for details; [59,60,61]). PS2 has two isoforms: isoform 1 is found in placenta, skeletal muscle and heart, while isoform 2, which lacks amino acids 263–296, is found in brain, heart, placenta, liver, pancreas, skeletal muscle, and kidney [56].

PSs have nine helical TM domains arranged with the hydrophilic, flexible N-terminus in the cytosol and the C-terminus protruding into the lumen of the extracellular space [62,63,64]. PSs form the catalytic core of γ-secretase [51,65,66,67]. This enzyme is indeed a high molecular weight heterotetrameric complex consisting of PS (PS1 or PS2), nicastrin, anterior pharynx defective 1 (APH1), and presenilin enhancer (PEN-2) (reviewed in [68,69]) that promotes intramembranous proteolysis of a variety of type I membrane proteins. Among them, there are APP, Notch, Delta1, E- and N-cadherins, CD44, Nectina-1α, ErbB4 and the β2 subunit of the voltage-dependent Na^+^ channel [70]. During the assembly and maturation of γ-secretase, the PS1, or PS2, subunit undergoes endoproteolytic cleavage into N- and C-terminal fragments (NTF and CTF, respectively), which then remain stably associated with each other [71,72,73]. NTF, of about 30 kDa, and CTF, of about 20 kDa, are generated from the immature holoprotein by an autocatalytic cleavage within the 7th hydrophobic domain (which is part of a large cytosolic loop) that occurs once PS is incorporated into the enzymatic complex forming a dimer. Although the cleavage of PSs is autocatalytic, PS maturation needs other molecular partners and represents a saturable process. The accumulation of non-mature form of PS, called full length (FL), leads to its proteasomal degradation. Because of this degradation, FL-PS has a very short half-life, around 1.5 hours, compared to the half-life of 24 hours of the mature form [74]. 

More than 150 autosomal dominant mutations in PS1 and 14 in PS2 are associated with FAD onset [75]. In agreement with the finding that PSs form the catalytic core of the γ-secretase, first studies showed that FAD-linked PS mutants are associated with increased activity, generating more Aβ peptides, in different cell models and tg mice carrying FAD-PS mutations [76,77,78]. This original idea has been challenged by other studies reporting, in FAD-PS expressing models, a lower total enzymatic activity, with an increase only in the Aβ42/Aβ40 ratio, due mainly to a drop in the production of Aβ40 [79,80,81]. Thus, although an intense debate is still ongoing on this topic, the first hypothesis of gain-of-function FAD-PS mutations has been revised into a loss-of-function view: FAD-PSs decrease total production of Aβ, due to the less precise cleavage of APP, altering the physiological balance of the process and causing an increased Aβ42 and a decreased Aβ40 generation. Recently, another mechanism has been proposed in which mutations in PS1 cause a deficiency in the carboxypeptidase function of γ-secretase affecting its trimming ability and thus favoring the production of Aβ42 [78,82].

In addition to their well-defined catalytic role within the γ-secretase complex, PSs are known to have pleiotropic γ-secretase-independent functions, mainly in regulating Ca^2+^ homeostasis, but also in protein trafficking, cell adhesion, and autophagy [73,83]. Multiple works by different groups showed that FAD-PSs, although by distinct mechanisms, alter some pathways of cellular Ca^2+^ homeostasis, contributing to the early phases of AD pathogenesis (see below). Moreover, the observation that SAD cases are also characterized by alterations in Ca^2+^ homeostasis led to the formulation of the “Ca^2+^ hypothesis” for AD, proposing a precocious and central role for Ca^2+^ in the pathogenesis of the disease. 

As mentioned above, PS2 is expressed not only in the brain but also in different tissues, indicating that the protein may have a role in non-neuronal cell processes. In particular, PS2 expression in the heart is needed for correct cardiac development, and the modulatory effects of the protein on calcium signaling have a role in cardiac systolic function ([84,85]). 

Importantly, mutations of PS genes have been described in Dilated Cardiomyopathy (DCM) and heart failure [86]. Over the past decades, intense efforts have been made to understand the relationship between cardiac defects and AD, but the issue remains still unclear [87,88]. An “heart-brain continuum” hypothesis has been made based on the fact that AD and some cardiac pathologies share many features, including common risk factors, similar protein aggregates and genetic mutations [87]. Indeed, protein aggregates found in the myocardium of idiopathic DCM patients are biochemically similar to brain AD deposits, and soluble pre-amyloid oligomers are commonly found in heart failure patients [89]. Importantly, abnormal protein oligomers/aggregates have been reported to disturb Ca^2+^ homeostasis and contribute to the cardiac pathology [90]. Thus, the common regulatory mechanism behind the convergence between AD and cardiac diseases could be the Ca^2+^ dysfunction [87] but the field is open to new hypotheses [91].

## 4. Ca^2+^ Molecular Toolkit and Signalling: A General Overview

**Figure 1:**
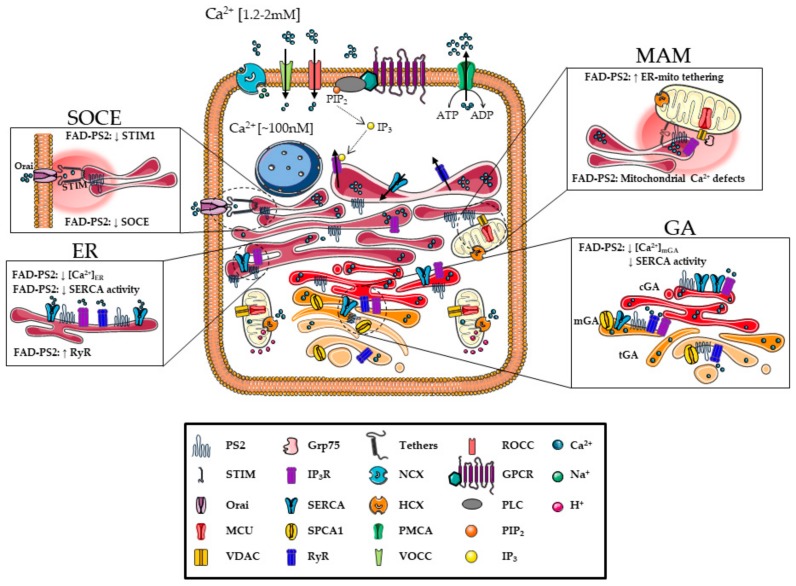
Ca^2+^ signaling pathways and molecular toolkit and their dysregulation by FAD-PS2. Cytosolic [Ca^2+^] ([Ca^2+^]_c_) can increase up to 1-3 μM upon cell stimulation thanks to a highly complex molecular toolkit that cooperates to maintain cell Ca^2+^ homeostasis. Ca^2+^ can cross the PM through different types of Ca^2+^ channels (ROCCs, SMOCCs, SOCCs, VOCCs) and/or it can be released from intracellular stores, primarily the endoplasmic reticulum (ER). Once the stores are depleted, a refilling process, called SOCE, occurs to restore their [Ca^2+^]. Extrusion mechanisms, i.e., PMCA and NCX in the PM, and re-uptake processes, such as that mediated by SERCA pumps, restore the resting [Ca^2+^]_c_. Finally, cytosolic Ca^2+^ rises can be modulated by mitochondria that can transiently and rapidly take up Ca^2+^ when Ca^2+^ hotspots are generated close to their surface, e.g., at MAM. PS2 can interact with many important elements of the Ca^2+^ toolkit thanks to its localization mainly in ER, GA (cis-, medial- and trans-GA) membranes and specific domains such as MAM. Boxes show specific FAD-PS-modulated Ca^2+^ pathways. See text for details.

Ca^2+^ plays a pivotal role in the regulation of multiple neuronal and astrocytic functions, from neurotransmitter release to synaptic plasticity [92], from membrane excitability to gene transcription, from proliferation to cell death [93]. All these different functions are orchestrated by a precise spatial and temporal regulation of Ca^2+^ concentration ([Ca^2+^]) that is ensured by a highly complex molecular Ca^2+^ toolkit composed of cell surface receptors, channels, pumps, antiporters, Ca^2+^ buffers, and Ca^2+^ sensors. They have specific distributions and roles within the cell, cooperating in the maintenance of cell Ca^2+^ homeostasis [94] (Figure 1). These components allow cells to maintain a large [Ca^2+^] gradient between the cytosol ([Ca^2+^]_c_ ~ 100 nM) and the extracellular medium ([Ca^2+^]_e_ ~ 1.2–2 mM), in resting conditions, and to increase the [Ca^2+^]_c_ up to 1–3 μM, upon cell stimulation. Indeed, Ca^2+^ can cross the PM and/or can be released from intracellular stores [95]. Different types of Ca^2+^ channels are present in the PM with a different distribution, resulting in a highly regulated connection between the intracellular and extracellular space: Receptor-Operated Ca^2+^ Channels (ROCCs); Second Messenger-Operated Ca^2+^ Channels (SMOCCs); Store-Operated Ca^2+^ Channels (SOCCs; see also below) or Voltage-Operated Ca^2+^ Channels (VOCCs) (Figure 1). VOCCs are particularly crucial in excitable cells as their opening is regulated by membrane depolarization. VOCCs have been classified into three families according to their voltage/inhibitor sensitivity: CaV1 (L-type), CaV2 (N-, P/Q- and R-type), and CaV3 (T-type). The N- and P/Q- types are present in synapses and control the Ca^2+^-dependent release of neurotransmitters; L-type are present in dendrites and soma and can mediate Ca^2+^-dependent gene transcription [96]. 

The other major source for the intracellular Ca^2+^ signal is the Ca^2+^ release from internal stores, mainly the ER. The extensive ER network presents two key intracellular Ca^2+^ releasing channels, the Ryanodine Receptor (RyR, see [97] for a recent review) and the Inositol 1,4,5-trisPhosphate (IP_3_) receptor (IP_3_R; see [98] for a recent review) families (Figure 1). By activation of phospholipase C (PLC), the Ca^2+^-mobilizing second messenger IP_3_ and diacylglycerol (DAG) are generated. IP_3_ interacts with IP_3_Rs, causing their opening and the release of Ca^2+^ from the lumen of the ER to the cytosol. Released cytosolic Ca^2+^ modulates, in a precise concentration-dependent manner, the IP_3_R opening and the activation, by direct binding, of RyRs, causing a further release of Ca^2+^ from the ER (Ca^2+^-induced Ca^2+^ release; CICR; Figure 1). Another important intracellular Ca^2+^ store is represented by the GA that partially shares the molecular Ca^2+^ toolkit with the ER [99,100], despite the fact that sub-compartments of the organelle, i.e., medial- and trans-GA, also express specific Ca^2+^ handling proteins (see below). Finally, secretory granules and lysosomes have been also proposed to be Ca^2+^-releasing organelles, possibly though Cyclic ADP-ribose (cADPR)-sensitive channels, but these mechanisms are still under debate (reviewed in [101]).

Once Ca^2+^ is released from the ER, the subsequent Ca^2+^ depletion induces a Ca^2+^ influx through highly specific PM channels, triggering a phenomenon called Capacitative Ca^2+^ Entry (CCE) or Store-Operated Ca^2+^ Entry (SOCE, reviewed in [102]). Crucial players of SOCE are STromal Interaction Molecule 1 (STIM1) and Orai1. Orai1 is the Ca^2+^ permeable channel at the PM, while STIM1 can “sense” the [Ca^2+^] in the ER lumen. After ER depletion, STIM1 changes its distribution forming clusters on ER membranes that localize close to the PM (puncta), interacting with, and opening, the PM-located Orai1 channels. This allows Ca^2+^ entry from the extracellular medium, increases in cytosolic [Ca^2+^] and refilling of empty stores [102] (Figure 1). Of note, despite STIM1 is the only protein required for SOCE activation, a similar protein called STIM2 plays a role in SOCE, maintaining the correct resting [Ca^2+^] in the ER lumen [103]. The ER-located Sarco/Endoplasmic Reticulum Ca^2+^ ATPase (SERCA) is responsible for the Ca^2+^ uptake of ER and cis/medial-GA, by pumping two Ca^2+^ ions in the organelle lumen for one ATP molecule consumed. There are three major paralogs, SERCA1-3, differentially expressed in different cell types, and additional post-translational isoforms of both SERCA2 and SERCA3 [104]. The most abundant form in the brain is SERCA2 [105]. The GA, in addition to SERCA, presents another pump for Ca^2+^ refilling, the Secretory Pathway Ca^2+^-ATPase 1 (SPCA1; [106]) (Figure 1). This pump is the only responsible for Ca^2+^ uptake in the trans-GA [106] and in part in medial-GA [107]. Importantly, Ca^2+^ store overfilling is prevented by the ER transmembrane protein TMCO1, acting as a Ca^2+^ Load Activated Ca^2+^ (CLAC) channel [108].

To maintain resting [Ca^2+^], and to restore cytosolic Ca^2+^ levels after Ca^2+^ rises, extrusion mechanisms, that transport Ca^2+^ out of the cell or back into intracellular stores, are necessary. Ca^2+^ is extruded from the cell by the PM Ca^2+^ ATPase (PMCA, reviewed in [109]) and the Na^+^/Ca^2+^ exchanger (NCX, [110]) (Figure 1). On the one hand, NCX represents the main Ca^2+^ extrusion system activated in excitable cells after a rise in cytosolic [Ca^2+^], due to its higher capacity and transport rate compared to PMCA [111]. These features enable NCX to function since the beginning of the recovery process, to rapidly remove large amounts of cytosolic Ca^2+^. On the other, PMCA, as well as SERCA, have lower capacity compared to NCX, but higher Ca^2+^ affinity, meaning that the pump plays a role later in the Ca^2+^ extrusion phase, completing the recovery process, to restore low resting cytosolic Ca^2+^ levels. 

The innumerable functions controlled by Ca^2+^ are guaranteed also by Ca^2+^-binding proteins [112] that can shape the Ca^2+^ signal in time and space through the rapid binding of the cation. Different Ca^2+^-binding proteins are present in both the cytoplasm and the organelle lumen. There are cytosolic Ca^2+^-binding proteins, such as calmodulin, parvalbumin, calbindin D-28k, and calretinin, as well as ER-located proteins, such as calsequestrin, calreticulin, glucose-regulated protein (GRP) 78, and GRP94 [93,113]. Other Ca^2+^ binding proteins are found in the lumens of GA and secretory vesicles [100]. 

Finally, mitochondria also play an important role in shaping cytosolic Ca^2+^ transients. Indeed, mitochondria are endowed with both Ca^2+^ uptake (the Mitochondrial Ca^2+^ Uniporter Complex, MCUC, [114,115]) and release (the Ca^2+^/Na^+^ antiporter, named NCLX [116], and the Ca^2+^/H^+^ antiporter [117]; Figure 1) mechanisms. Under resting conditions, the mitochondrial Ca^2+^ uptake and release mechanisms are in perfect kinetic equilibrium, which maintains the matrix [Ca^2+^] at levels comparable to those of the cytosol. When cytosolic Ca^2+^ levels increase, thanks to MCUC [114,115], Ca^2+^ is rapidly accumulated within the matrix. Ca^2+^ is only transiently sequestered into mitochondria as, when cytosolic Ca^2+^ levels start to decrease, the cation is released back to the cytosol by the Na^+^/Ca^2+^ and the H^+^/Ca^2+^ exchangers (Figure 1). It is worth noting that, the MCUC Ca^2+^ affinity (~20 µM) largely exceeds the [Ca^2+^] reachable in the cytosol upon cell stimulation (2–3 µM). This implies that mitochondria can take up Ca^2+^ very rapidly only when microdomains of high [Ca^2+^] occur close to their surface. These Ca^2+^ microdomains are typically formed near the mouth of channels and receptors permeable to Ca^2+^. Thus, the mitochondria that are able to rapidly take up Ca^2+^, during cytosolic Ca^2+^ rises, are those in close proximity to PM Ca^2+^ channels or ER Ca^2+^ releasing channels [118]. It needs to be stressed that increases in intra-mitochondrial Ca^2+^ levels occur for any rise of cytosolic [Ca^2+^], but these increases are slow and small for organelles distant from the microdomains. In particular, sites of proximity between ER and mitochondria constitute specific subcellular regions, called MAM (for a recent review see [119]; Figure 1), in which Ca^2+^ microdomains can easily form. These ER membrane domains are highly dynamic structures that can change in number and extension upon specific cell stimulations and needs. ER-mitochondria juxtapositions are particularly important in cell pathophysiology as they are believed to be not only the main sites of constitutive Ca^2+^ shuttling between the two organelles but hosting also crucial players in lipid biosynthesis, inflammation, autophagy and apoptosis. Indeed, various alterations in MAM domains have been linked to pathological conditions, such as cancer, neurodegenerative diseases, and metabolic syndromes (reviewed by [120]).

## 5. Presenilin 2 and Ca^2+^ Homeostasis

In 1988, Khachaturian firstly proposed that sustained alterations in Ca^2+^ homeostasis could be responsible for AD neurodegeneration [121]. Altered cytosolic Ca^2+^ responses have been found in peripheral cells from SAD or FAD patients [122,123]. Lately, dysregulation of Ca^2+^ handling has been reported in different AD mouse models (reviewed in [124]), as well as in aged and diseased brains (reviewed in [125]). mRNA levels of several genes involved in the maintenance of Ca^2+^ homeostasis have been found to be altered in cerebral tissues from AD patients [126], further sustaining a link between Ca^2+^ signaling defects and the pathology. Finally, an imbalance in Ca^2+^ handling has been proposed as an early event in the pathogenesis of FAD [127,128,129], although the mechanisms through which FAD-PS mutants affect Ca^2+^ dynamics are still controversial. 

Originally, studies in fibroblasts from asymptomatic FAD-PS patients reported an increased IP_3_-mediated Ca^2+^ release from the ER [127,128]. These studies lay the groundwork for the formulation of the “Ca^2+^ overload” hypothesis for AD that sustains that FAD-linked PS mutations, by increasing ER Ca^2+^ content, cause excessive Ca^2+^ release in the cytosol, altering APP processing, increasing neuronal sensitization to Aβ and producing cytotoxic stimuli that eventually lead to a Ca^2+^-dependent cell death [130]. This hypothesis has been further supported by evidence of an increased activated ER Ca^2+^ release in different FAD-PS1 cell models and neurons from FAD-PS1 tg mice [131,132,133]. Furthermore, other data showed that FAD-PS1 and FAD-PS2 mutations induce the potentiation of ER Ca^2+^ release from both RyRs [132,134,135,136,137,138,139] and IP_3_Rs [140,141,142]. Finally, it has been reported that wild type (wt) PS holoproteins form passive Ca^2+^ leak channels in planar lipid bilayers and FAD-PS mutants impair this leak activity, leading to an increased ER Ca^2+^ content [143,144,145]. 

The initially proposed “Ca^2+^ overload” hypothesis, however, has been later challenged by several groups, reporting conflicting data on ER Ca^2+^ overload in the presence of FAD-PSs, suggesting alternative explanations for the old data and opening a new scenario. In particular, an increase in ER Ca^2+^ release has been reported in the absence of an ER Ca^2+^ overload, due to the hyperactivity of IP_3_Rs [146,147]. Moreover, multiple FAD-linked PS1 mutations showed no effect on [148], or even attenuated [149], ER Ca^2+^ levels. Importantly, additional studies did not confirm the hypothesis that PS1 holoprotein functions as an ER Ca^2+^ leak channel [150]. On the same line, several FAD-linked PS2 mutations have been shown to reduce, instead of increasing, the cytosolic Ca^2+^ rise induced by an IP_3_-generating agonist (or by inhibiting SERCA pumps), in both fibroblasts from FAD patients and cell lines stably or transiently expressing the PS2 mutant [60,129,151,152,153] (see also below and Figure 1). Thus, the more recent view on Ca^2+^ dysregulation in AD agrees with a Ca^2+^-based neuronal hyperexcitability, yet in the presence of unmodified or reduced ER Ca^2+^ content. It is worth noting that the above described discording results on ER Ca^2+^ content obtained in multiple FAD-PS models could be due to the different specificity of the probes used to measure Ca^2+^ dynamics (see Box 1). Indeed, most of the recent studies questioning the Ca^2+^ overload hypothesis has been performed employing Genetically Encoded Ca^2+^ Indicators (GECIs), specifically targeted to the lumen of different intracellular Ca^2+^ stores (see Box 1), thus measuring directly their Ca^2+^ content. Instead, classical cytosolic chemical dyes give an indirect (and sometimes misleading) estimation of stored Ca^2+^ by measuring the amplitude of the cytosolic Ca^2+^ rises upon cell stimulations. 

The involvement of PSs in regulating ER Ca^2+^ homeostasis is further supported by studies that showed a physical interaction of PSs with sorcin [154], a protein regulating RyR activity, calsenilin [155], and calbindin D28K [156], two Ca^2+^ buffer proteins, SERCA2b [157], IP_3_Rs [146], and RyRs [137]. Furthermore, PSs result enriched in MAM, the specific ER membrane domains in close apposition with mitochondria [59,60,61,158]. However, only PS2, and not PS1, acts as a positive modulator of ER-mitochondria tethering and Ca^2+^ transfer [60,61,158], with FAD-PS2 mutants more effective in this function compared to the wt molecule, suggesting a pathogenic effect of mutants on this signaling axis (Figure 1). An alternative explanation is that the increased number of contact sites between ER and mitochondria, observed in FAD-PS2 cells, compensates for the decrease in ER [Ca^2+^] caused by mutated PS2, augmenting the number of Ca^2+^ microdomains on organelle surface, thus increasing the efficiency of ER to mitochondria Ca^2+^ transfer [60,152,159]. Interestingly, an increased ER-mitochondria coupling has been observed in several AD cellular and animal models, in cells acutely expose to Aβ oligomers, as well as in diseased human brains [160], reinforcing the idea that an alteration in ER-mitochondria connection represents a common feature in neurodegeneration [161]. 

As mentioned above, FAD-PS2 mutants reduce ER Ca^2+^ content and the cytosolic Ca^2+^ rise induced by stimuli that cause Ca^2+^ store depletion [60,129,148,151,152,153,159,162,163] (Figure 1). This effect is due to the capacity of the protein to interfere with SERCA activity, partially blocking it [162]. More recently, the effect of FAD-PS2 on Ca^2+^ handling has been tested not only in the ER, the main intracellular Ca^2+^ store, but also in other Ca^2+^ storing organelles, such as the GA. In particular, Ca^2+^ homeostasis was investigated in the medial- and trans-GA by two specifically targeted FRET-based Ca^2+^ probes (see Box 1). The analysis indicates that FAD-linked PS2 mutations decrease the Ca^2+^ content of the medial-GA, without affecting the trans-GA [153] (Figure 1), extending the results obtained earlier employing an aequorin Ca^2+^ probe targeted to the whole GA [148]. The ineffectiveness of PS2 on trans-GA is due to the fact that this sub-compartment does not express, as Ca^2+^ refilling mechanism, SERCA pumps, whose activity is inhibited by FAD-PS2 [162], but only SPCA1 [106]. The medial-GA, instead, being endowed with both pumps [107], results partially depleted in its Ca^2+^ content by FAD-PS2 expression [153]. Of note, FAD-linked PS1 mutants do not cause significant changes in ER and GA Ca^2+^ levels [148,153], as well as in ER-mitochondria tethering [60,152,158]. 

The data summarized so far suggest a differential role of PS1 and PS2 in Ca^2+^ homeostasis regulation. However, both FAD-PS1 and FAD-PS2 mutants present also similar features related to Ca^2+^ handling: they both increase the expression of RyRs [159,164,165] and induce hyperactivity of IP_3_Rs [146] and they both reduce SOCE. The decreased SOCE has been found in different cell models expressing PS1 or PS2 mutants [129,133,148,151,153,166,167], as well as in human fibroblasts from both PS1 and PS2 FAD-patients [129,153] (Figure 1). This effect of PSs on SOCE is explained, at least partially, by a significant reduction in STIM1 levels, found in mutant PS-expressing cells, human FAD fibroblasts, and in the brains of SAD patients [153,168]. Importantly, a SOCE attenuation has been reported also in mushroom spines of hippocampal neurons from FAD-PS1 M146 V knock-in mice, where STIM2 seems to be the essential SOCE component responsible for the degeneration of neuronal spines [169,170]. If the PS-mediated SOCE effect is dependent or not on γ-secretase activity is still under debate [151,153,166,171,172]. On the other hand, the effects of mutants PSs on intracellular Ca^2+^ stores appear to be independent of γ-secretase activity [151]. Indeed, in cells KO for both PSs, the expression of the PS2-D366A mutant (a loss-of-γ-secretase-function mutant of PS2 that precludes its endoproteolysis, preventing its incorporation in the γ-secretase complex) has been shown to cause effects on Ca^2+^ handling similar to those reported in FAD-PS2 mutant-expressing cells [151]. Moreover, in the same cells, the co-expression of PS2 NTF and CTF, which recovered the γ-secretase activity in these cells, failed to mimic the effect of wt or mutant PS2 on Ca^2+^ handling [162], indicating that the PS2 active form in Ca^2+^ homeostasis is the holoprotein [162].

Of note, Ca^2+^ dysregulation caused by FAD-PS2 was also found in primary neurons, the most suitable AD cell model. Indeed, these cells represent the primary target of the disease and neuronal Ca^2+^ homeostasis is fundamental in sustaining neuronal activity. Taking advantage of PS2.30H and B6.152H PS2-based tg mice (see Box 2), expressing the PS2-N141I mutation in the absence, or presence, of the APP Swedish (APPswe) mutation, respectively, it was confirmed that FAD-PS2 causes a decrease in ER Ca^2+^ content in both cortical neuronal cultures and hippocampal brain slices. The altered Ca^2+^ phenotype has been found in both tg lines, indicating that PS2 mutations are sufficient per se to cause Ca^2+^ dysregulation (in particular, to reduce ER Ca^2+^ content [159], as revealed by employing a FRET-based Ca^2+^ probe targeted to the ER lumen, the D4ER, [173]; see Box 1). The effect of FAD-PS2 on ER-mitochondria tethering and Ca^2+^ transfer was confirmed in these neurons. Furthermore, neurons and hippocampal slices from both tg lines show an increase in Ca^2+^ excitability, confirming that the PS2-N141I mutation alone (without the additional APP mutation-linked effect, i.e., a substantial increase in Aβ peptide production) is sufficient to cause Ca^2+^ hyperactivity and ER/mitochondria Ca^2+^ dysregulation [159]. 

Alterations in Ca^2+^ homeostasis due to FAD-linked PS2 mutations have multiple consequences on cell physiology, including impaired autophagy [174,175] and defective mitochondrial activity [176,177]. Moreover, in primary neurons from B6.152H tg mice, cell bioenergetics was also significantly affected [178]. Finally, the Ca^2+^ hyperactivity described in both FAD-PS2 mouse models likely contributes to the hyper-excitability found at the brain network level in adult, anesthetized animals, in the presence or absence of Aβ accumulation [163,179,180]. 

Ultimately, a possible link between the “amyloid cascade” and the “Ca^2+^ hypothesis” in AD pathogenesis emerged from studies showing that, firstly, Ca^2+^ dysregulation modulates amyloid plaque formation and secondly, amyloid plaques and soluble Aβ aggregates increase cytosolic [Ca^2+^], by different mechanisms (reviewed in [124]).

Importantly, often the overexpression of the wt PS2 mimics the effect of FAD-PS2 mutants on Ca^2+^ homeostasis [153]. However, Ca^2+^ dysregulation seems to correlate with PS2 protein expression, and higher levels of wt PS2, compared to those of FAD mutants, are required to obtain similar effects on Ca^2+^ handling [129]. Thus, it can be speculated that the effect on Ca^2+^ signaling could be due to an accumulation of PS2 (wt and FAD) holoprotein (see above). Importantly, higher levels of wt PS2 have been found also in several SAD brain samples, due to the loss of the transcription factor REST (Repressor Element 1-Silencing Transcription factor). REST is a neuronal repressor of transcription that modulates different genes, including *PSEN2*, promoting cell death and AD [181]. Thus, the loss of REST causes an up-regulation of wt PS2 transcript/protein that in SAD could mimic the Ca^2+^ dysregulation effects of FAD-PS2 mutants. 

## 6. Concluding Remarks

PS1 and PS2 mutations account for more than 80% of the genetic lesions responsible for FAD. The two proteins represent the catalytic core of the γ-secretase complex that, by cleaving APP in concert with β-secretase, produces neurotoxic Aβ peptides involved in AD pathogenesis, in accordance with the so-called amyloid cascade hypothesis. However, the mechanism linking these mutations to neuronal dysfunction, and eventually cell death, is still largely obscure and the pharmacological targeting of Aβ production/accumulation currently failed to halt AD. 

Many studies performed in different FAD models indicate alterations in Ca^2+^ signaling as early events in the pathology, despite contrasting results on how Ca^2+^ handling is modified have been reported. Nowadays, it is well known that Ca^2+^ homeostasis controls different processes required for normal brain function, such as neurotransmitter release and memory and learning processes, as well as different detrimental processes (e.g., cell death and degeneration [93,182]). The current and common idea is that in AD an altered neuronal Ca^2+^ handling, due to excessive Ca^2+^ release from intracellular stores (mainly ER through its Ca^2+^ releasing channels IP_3_Rs and RyRs), causes neuronal Ca^2+^ hyper-excitability, independently on the Ca^2+^ content of the ER. On the other hand, however, ER (and GA) Ca^2+^ depletion can cause, in the long term, additional problems. For example, as the ER and mitochondria are physically and functionally coupled, ER Ca^2+^ alterations affect mitochondrial Ca^2+^ signaling, possibly leading to metabolic defects that culminate in neuronal death activation. In this view, FAD-PS2 mutants, by reducing ER and medial-GA Ca^2+^ content and increasing ER-mitochondria apposition, play a key role in AD-linked altered Ca^2+^ homeostasis. Moreover, PS2 and PS1 have also been shown to share some Ca^2+^ related phenotypes, such as the negative impact on SOCE and the increased expression levels of RyRs, linked to a potentiated Ca^2+^ release from the ER. Based on this evidence, a mitochondrial Ca^2+^ dysregulation can be proposed as a convergent pathological pathway between PS1- and PS2-dependent AD, linking defective global Ca^2+^ signals, organelle dysfunction and neurodegeneration. Indeed, either a mitochondrial Ca^2+^ overload, caused by an excessive release of Ca^2+^ from ER or by neuronal Ca^2+^ hyperexcitability, or a reduced mitochondrial Ca^2+^ signal, unavoidably linked to impaired ATP production, caused by a decreased ER Ca^2+^ content or a reduced SOCE, could be no-return signals that trigger neuronal cell death. 

### 6.1. BOX1. Calcium Imaging Tools

**Figure 2:**
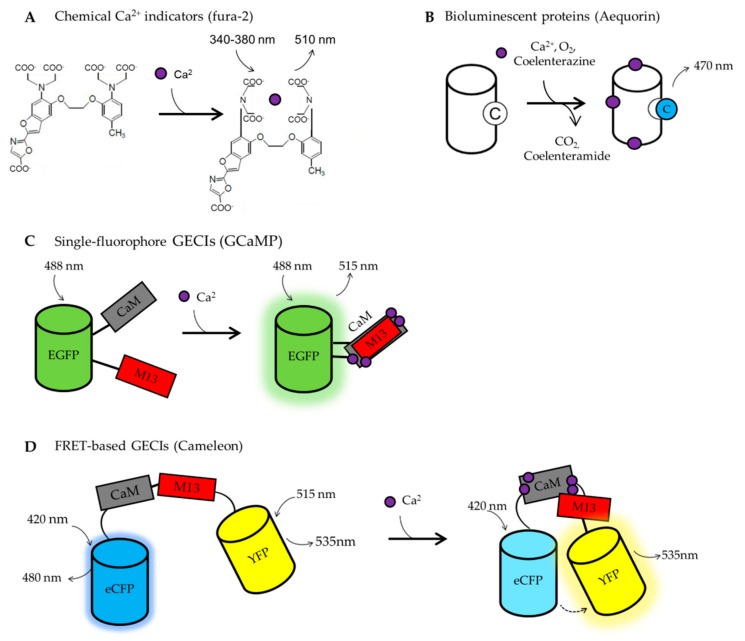
Indicators. (**A**) Chemical calcium indicators: fura-2. Following the binding of Ca^2+^ to fura-2, the probe emission, at 340 nm of excitation, increases, whereas that at 380 nm decreases. The 340/380 ratio is dependent on Ca^2+^ concentration. (**B**) Bioluminescent proteins: Aequorin. When Ca^2+^ binds aequorin, the prosthetic group of coelenterazine (**C**, left side) oxidates to coelenteramide (**C**, right side), emitting a photon at 470 nm. (**C**) Single-fluorophore-based GECIs: GCaMP. Binding of Ca^2+^ to GCaMP leads to conformational changes between the two Ca^2+^ responsive elements that increase the emitted fluorescence. (**D**) FRET-based GECIs: Cameleon. Binding of Ca^2+^ to CaM, induces a conformational change between CaM and M13 that enables FRET between donor and acceptor fluorophores.

Ca^2+^ indicators are fluorescent or luminescent molecules that undergo spectral modifications following Ca^2+^ binding (Figure 2). These changes permit the recording of free [Ca^2+^] in living cells, in real-time. Tsien and colleagues created the first generation of synthetic, chemical probes [183], fusing a fluorescent dye with Ca^2+^-selective chelators (EGTA or BAPTA). Among these chemical probes, fura-2 [184], and in particular its cell-permeant version containing the acetoxymethylated ester (AM) group, is the most used to monitor [Ca^2+^]_c_ [185]. Fura-2 is a ratiometric indicator with a dissociation constant (K_d_) of 140 nM in vitro. It is excited at 340 nm and 380 nm and it emits at 510 nm (Figure 2A). The emission ratio is dependent on [Ca^2+^]. Indeed, upon Ca^2+^ binding, the emission at 340 nm of excitation increases, whereas the emission at 380 nm of excitation decreases. The ratiometric properties of this dye allow accurate measurements of Ca^2+^ dynamics in living cells, being independent on dye leakage, photobleaching, differential dye loading, focal plane changes or cell movement artifacts. These parameters cannot be controlled using non-ratiometric probes, such as Fluo-4. A limitation of the classic fura-2, and of chemical probes in general, is that it cannot be specifically targeted to intracellular organelles. However, recently, this limitation has been overcome by the development of a mitochondria-targeted version of fura-2, the mito-fura, with a K_d_ of 1.5 μM in vitro and 6 μM in living cells [186]. The different affinities for Ca^2+^, displayed by mito-fura in vitro and in cells, indicate that, for quantitative measurements, a precise probe calibration in the biological system under investigation is required. 

A revolution in the field of Ca^2+^ imaging is represented by the development of Genetically Encoded Calcium Indicators (GECIs) (Figure 2B–D). Being genetically encoded, GECIs can be targeted to different subcellular compartments and their expression can be controlled both spatially and temporally. The first developed GECI was based on aequorin (Aeq, [187]) (Figure 2B) and only later GECIs based on the Green Fluorescent Protein (GFP) have been generated [188] (Figure 2C,D). 

Aeq is a Ca^2+^ sensitive bioluminescent protein extracted from the jellyfish, *Aequoria victoria* [189]. It consists of an apoprotein, named apoaequorin, that requires the luminescent substratum coelenterazine to function. Upon Ca^2+^ binding, coelenterazine is irreversibly oxidized to coelenteramide, emitting a photon at 470 nm. The rate of photon emission is proportional to [Ca^2+^] (Figure 2B). The large dynamic range, the low interference with endogenous Ca^2+^ buffering proteins and the low sensibility to pH make Aeq-based indicators good sensors for cell population analysis. However, Aeq suffers from some disadvantages, i.e., a low emitted light and the consumption of the probe during the experiment, making this GECI mainly suitable for relatively short (tens of minutes) experiments in cell populations [190]. Nowadays, several modified Aeq-based probes, with different Ca^2+^ affinities and targeted to almost all the subcellular compartments, are available (see [191] for a complete list). The wt Aeq can report [Ca^2+^] dynamics between 1–10 μM, the mutated form (D119A, [192]) can sense variation from 10 to 100 μM and the ER-targeted versions (N28L, [193,194]) are able to work in the mM [Ca^2+^] range. Being able to sense Ca^2+^ in the μM range, Aeq does not suffer from buffering capacity problems, meaning that its usage does not interfere with the physiology of Ca^2+^ signaling.

The GFP-based GECIs are the most used as they are easy to use and allow a single-cell analysis of real-time Ca^2+^ dynamics. Generally, they consist of a Ca^2+^ binding domain fused to a fluorescent protein (FP). When the Ca^2+^ ions bind the specific responsive domain, the FP changes its fluorescent properties. The use of fluorescent GECIs presents numerous advantages over chemical and bioluminescent Ca^2+^ sensors, such as the possibility of monitoring changes in [Ca^2+^] in real-time within specific organelles, restricting the analysis only to co-transfected cells expressing also the protein under investigation. They can be divided into two main groups: single fluorophore-based GECIs (Figure 2C) and Förster resonance energy transfer (FRET)-based GECIs (Figure 2D). 

The first group includes camgaroos, pericam, and GCaMPs family. They are characterized by the presence of an FP, typically circularly permuted, and a Ca^2+^ binding domain inserted within the FP sequence, nearby the chromophore. Thus, changes in intensity or emission wavelength occur upon Ca^2+^ binding (Figure 2C). Among the single fluorescence-based GECI, GCaMPs [195] are the most used. They have been generated from the fusion of a circularly permuted GFP with two Ca^2+^ responsive elements: calmodulin (CaM) and a peptide sequence from the myosin light chain kinase (M13) (Figure 2C). These sensors are highly sensitive to Ca^2+^, with a K_d_s from 0.15 to 2 μM depending on the variants in use. These K_d_s have been evaluated in vitro and, as far as we know, in situ calibrations are missing. Of note, non-ratiometric sensors are difficult to calibrate being their fluorescence affected by changes in both cell morphology and focal plane. One of the last versions, i.e., GCaMP6 [196], has been widely used in the neuroscience field to study Ca^2+^ dynamics in different cell sub-compartments in living organisms. Indeed, many tg mouse lines expressing these specifically targeted probes have been created [197], allowing researchers to extend their investigation in vivo [198,199]. However, the recently observed neurotoxicity upon chronic expression of GCaM6P [200,201] imposes caution in the usage of such tg mouse lines, suggesting that GECI viral delivery or inducible/tissue-specific expression are preferable over their ubiquitous and constitutive expression. This toxic side effect could be partially explained by the fact that being these probes highly sensitive to Ca^2+^, they have a high Ca^2+^ buffering capacity that can interfere with various physiological processes when chronically expressed.

The second group of GFP-based GECIs are represented by those based on FRET, such as Cameleon probes [202,203], or their variants based on Troponin C, as Ca^2+^ responsive element [204]. Among the FRET-based GECIs, Cameleons are the first generated and the most used (Figure 2D). They have been targeted to many intracellular organelles, allowing researchers to analyze organelle Ca^2+^ dynamics in living cells. They are based on cyan (CFP) and yellow (YFP) variants of GFP and they exploit M13 and CaM for sensing Ca^2+^. Briefly, Ca^2+^ ions bind to CaM causing a conformational change that allows M13 to wrap around CaM, forcing the two FPs closed by. These changes in the molecule’s structure allow FRET to occur between the two fluorophores (Figure 2D). Thus, upon Ca^2+^ binding, the CFP fluorescence decreases, whereas the YFP fluorescence increases. Therefore, the YFP/CFP emission ratio changes dependently on [Ca^2+^]. Cameleon probes have all the advantages of other ratiometric probes [205]. Nowadays, many variants are available with K_d_s from 0.1 to almost 70 μM in vitro. Many Cameleon K_d_s have been also calculated in living cells and are reported in [206].

### 6.2. BOX2. PS2-Based Mouse AD Models

Numerous tg mouse lines expressing wt or mutated human APP and/or PS have been generated to provide animal models of AD. The expression of human FAD mutant of PS1 or PS2 in mice does not cause the characteristic histological hallmarks found in AD patients as amyloid plaques or neurofibrillary tangles are absent. This is because the mouse APP is not-amyloidogenic. Generally, *PSEN* mutations accelerate plaque formation in double tg lines expressing both FAD *PSEN* (*PSEN1* or *PSEN2*) and human FAD *APP* mutated genes (mainly APPswe, carrying two amino acidic mutations in position 670 and 671) [207,208,209]. A complete list of the available AD mouse models is provided in [210].

The first-generation mouse models, based on APP or APP/PS overexpression, present however some limitations, i.e., overproduction of APP fragments in addition to Aβ [211] and lack of a clear phenotype standardization of the different models [212]. To overcome these limitations, second-generation mouse models were created. These new mice have been obtained by an *APP* knock-in strategy and present overproduction of Aβ42 without APP overexpression. Like the first-generation mouse models, also these mice present limitations, i.e., they do not exhibit tau pathology or neurodegeneration (see [211] for further details).

Focusing on PS2 and APP, the homozygous tg lines PS2-N141I (PS2.30H) and PS2APP are commonly used. The PS2-N141I (PS2.30H) mouse line expresses the human *PSEN2* gene carrying the N141I mutation under the control of the mouse prion protein promoter [213]. This model is still poorly characterized since it has been created only to generate double tg PS2APP mice. Two PS2APP mouse models are present in literature: the first refers to the model generated by co-injecting two constructs into C57/BL/6 zygotes, known as B6.152H [214]. The other PS2APP line was generated by crossing two single tg animals (PS2N141I x APPswe) [213]. In this latter model, the human APPswe expression is controlled by the Thy1.2 promoter. Both lines of double tg animals express the same transgenes and both develop a similar level of cerebral pathology [215], but only B6.152H mice show enhanced LTP (see below). B6.152H mice have higher expression of tg human *APP* mRNA (1.45-fold) compared to PS2APP mice, and approximately 30-fold higher levels of *PSEN2* mRNA. They also have about 2-fold higher Aβ levels than PS2APP mice [216]. The B6.152H line has been used also to generate the triple tg line, TauPS2APP [217], in which a mutation in Tau (P301L) is also present. 

In B6.152H mice, amyloid plaques are absent at 3 months of age. The overt Aβ deposition in the brain appears only at approximately 6 months, with heavy plaque load in the hippocampus, frontal cortex, and subiculum at 10 months. Aβ deposits in blood vessels were observed sporadically and mainly in large vessels. The amount of cerebral amyloid deposits correlated with levels of the human *APP* transcript at 12 months [214,215]. Decreased survival of new-born neurons in the dentate gyrus, at about 4 months compared to wt, has also been observed [216]. Furthermore, a strong increase in LTP and post-tetanic potentiation (PTP) in hippocampal slices of 10-month-old animals, compared to wt mice, has been reported [216]. Finally, a decreased perfusion of the occipital cortex occurs from 10 to 17 months of age in these tg mice [215]. 

In general, despite several AD mouse models have been generated, none of them fully recapitulates the human pathology [211,218], strongly indicating that further efforts should be devoted to generating a model that can better mimic both SAD and FAD disease progression.
